# Large-Area Pulsed Laser Deposition Growth of Transparent Conductive Al-Doped ZnO Thin Films

**DOI:** 10.3390/nano15221722

**Published:** 2025-11-14

**Authors:** Elena Isabela Bancu, Valentin Ion, Mihai Adrian Sopronyi, Stefan Antohe, Nicu Doinel Scarisoreanu

**Affiliations:** 1 National Institute for Laser, Plasma and Radiation Physics (INFLPR), Atomistilor Street 409, 077125 Magurele, Romania; elena.bancu@inflpr.ro (E.I.B.); valentin.ion@inflpr.ro (V.I.); mihai.sopronyi@inflpr.ro (M.A.S.); 2 Faculty of Physics, R&D Center for Materials and Electronic & Optoelectronic Devices (MDEO), University of Bucharest, Atomistilor Street 405, 077125 Magurele, Romania; santohe@solid.fizica.unibuc.ro; 3 Academy of Romanian Scientists (AOSR), Ilfov Street 3, 050045 Bucharest, Romania

**Keywords:** PLD, large area, AZO, TCO

## Abstract

High-quality AZO thin films were produced on a 4-inch Si substrate using large-area PLD equipment at a substrate temperature of 330 °C, with a ZnO: Al (98:2 wt.%) target. This study aims to enhance the electrical, optical, morphological and structural properties of large-area PLD-grown AZO thin films by tuning the deposition pressures. The samples were prepared under high-vacuum (HV) conditions, as well as in oxygen atmospheres of 0.005 mbar O_2_, 0.01 mbar O_2_, and 0.1 mbar O_2_. Consequently, a bilayer AZO film was prepared in a combination of two deposition pressures (first layer prepared under HV, followed by the second layer prepared at 0.01 mbar O_2_). Additionally, morphological and structural characterization revealed that high-quality columnar growth AZO thin films free of droplets, with a strong (002) orientation, were achieved on a 4-inch Si substrate. Moreover, Hall measurements in the Van der Pauw configuration were used to assess the electrical properties. A low electrical resistivity of 3.98 × 10^−4^ Ω cm, combined with a high carrier concentration (n) of 1.05 × 10^21^ cm^−3^ and a charge carrier mobility of 17.9 cm^2^/V s, was achieved at room temperature for the sample prepared under HV conditions. The optical characterization conducted through spectroscopic ellipsometry measurements showed that the large-area AZO sample exhibits an increased optical transparency in the visible (VIS) range with a near-zero extinction coefficient (k) and a wide bandgap of 3.75 eV, fulfilling the standards for materials classified as TCO. In addition, the increased thickness uniformity of the prepared AZO films over a large area represents a significant step in scaling the PLD technique for industrial applications.

## 1. Introduction

Transparent conductive oxides (TCOs) are commonly used as front electrodes in a wide range of optoelectronic devices. These layers provide a unique combination of low resistivity and high optical transparency, mainly achieved by tuning the intrinsic properties. Additionally, an available TCO should exhibit an optical transmittance exceeding 80% across the VIS spectrum, while its electrical resistivity must reach values around 10^−4^ Ω cm [1,2]. Nowadays, Indium Tin Oxide (ITO) is the most widely used TCO in various applications, especially in the display market [3]. However, ITO is expensive mainly due to the limited availability of indium (In), and its price continues to rise in response to the rapid growth of photovoltaic (PV) and display industries [4]. Another drawback of ITO is its requirement for high processing temperatures since it exhibits poor structural quality at low temperatures [5], which restricts its applications in flexible devices. This limitation mainly arises from the limited tolerance of conventional PET substrate at high processing temperatures [6]. Another limitation is related to the diffusion of In ions into the active layer of organic photovoltaic solar cells (OPVs) during operation, which compromises the performance of the devices [7]. Furthermore, ITO exhibits poor stability in hydrogen plasma, where the oxide surface is reduced to its elemental constituents, which in turn affects the optical properties of the films in the VIS and IR regions [8]. Moreover, the instability of ITO in hydrogen plasma classifies it as incompatible with Si:H (silicon heterojunction) solar cells [9]. Another alternative to ITO was fluorine-doped tin oxide (FTO). However, the issue related to the high processing temperature required to achieve good optical and electrical properties has limited its application in flexible devices [10].

Among the available materials, a viable solution is the indium-free TCO based on aluminum-doped zinc oxide (AZO). The advantages of AZO include cost-effectiveness, abundant resources, and low toxicity, combined with chemical and mechanical stability [1,11]. Additionally, this material is an excellent choice for TCO applications as it possesses a wide band gap (3.37 eV), high binding energy (60 meV), and high transmittance across the visible spectrum (80%), while maintaining low electrical resistivity (10^−4^ Ω cm) [2,12,13,14]. The conductive behavior of ZnO arises from two principal mechanisms: first, intrinsic defects, including oxygen vacancies (V_o_), or zinc interstitials (Zn_i_), and second, extrinsic doping with group III elements such as Al, Ga, or In. Nevertheless, controlling intrinsic defects does not significantly improve the electrical conductivity of ZnO films. Additionally, at higher temperatures, the samples are unstable since the oxygen-deficient sites tend to reoxidize [15]. However, the role of native defects should be considered. According to A. F. Kohan et al. [16], oxygen vacancies and Zn interstitials are the most abundant defects in ZnO, which are also associated with improved electrical properties. On the other hand, the substitution of Zn^2+^ ions with Al^3+^ ions substantially increases the free-carrier concentration by adding one extra electron, since Al has a higher valence than Zn [17]. Regarding the doping concentration over a large area, Barbara Swatowska et al. [18] reported that optimal electrical and optical properties are achieved at doping concentrations of 2% and 3%. In contrast, for concentrations above 3% the resistivity of the sample starts to increase. Additionally, an increase in carrier concentration will lead to a degradation of the electrical properties, acting as scattering centers at grain boundaries, thereby increasing the electrical resistivity [19]. This occurs because at low doping concentrations, Al ions act as dopants, increasing the free-carrier concentration in TCOs, while at high doping concentrations, they act as impurities, leading to an increase in resistivity in TCOs [20].

Since substrate temperature plays a crucial role in flexible electronic devices, a recent study conducted by V.O. Anyanwu et al. [21] investigated the deposition of AZO thin films by PLD at low processing temperatures. Their study indicates that optimal results are achieved at a processing temperature of 150 °C, with an average transmittance exceeding 83% in the measured region and an electrical resistivity of 6.8 × 10^−5^ Ω cm. Considering the impact of oxygen pressure on the principal properties of TCOs, a recent study conducted over a large area by Amol C. Badgujar et al. [22] reported that a low oxygen flux facilitates the formation of native defects, including oxygen vacancies and metal interstitials, reducing the electrical resistivity. In contrast, they showed that high oxygen flux enhances transmittance but also increases electrical resistivity. Additionally, Ke Sun et al. [23] demonstrated that tuning the bandgap of ITO thin films by Al doping and thermal annealing improves the optical and electrical properties of ITO:Al layers, since the Al doping is responsible for an increased band gap, which enhances the transmittance in the UV region. The ITO:Al TCO was employed as a p-electrode in a blue light-emitting diode (LED) device fabricated over a 4-inch area, improving the device characteristics.

In terms of manufacturing, AZO films can be produced using various techniques, including RF and DC magnetron sputtering, atomic layer deposition (ALD), spray pyrolysis, and pulsed laser deposition (PLD) on both small and large-area substrates. Among the deposition methods used to prepare AZO thin films, magnetron sputtering is the most common as it ensures the production of coatings over large areas [24]. Still, one of the lowest electrical resistivities, at 8.5 × 10^−5^ Ω cm, was achieved using PLD [25]. On the other hand, PLD is recognized as a simple and versatile method for producing oxide thin films with high structural quality, good adhesion, and a stoichiometry close to that of the target [26,27]. However, problems related to depositions over large areas, together with reduced film uniformity, limit the upscaling of this technique. An important aspect in producing AZO through PLD is controlling the structural and electrical properties by adjusting the gas atmosphere and substrate temperature [28]. Additionally, placing the laser source outside the deposition chamber allows for greater flexibility in tuning the processing parameters, such as spot size or beam scanning [29]. Moreover, the pulsed nature of the laser allows for precise control over the thickness. Nonetheless, PLD is often considered challenging to scale up in industrial applications, mainly due to the anisotropic nature of ablation, which leads to non-uniformity in both thickness and composition over large areas [4,30]. The uniformity challenge mainly arises from the mismatch between the large area coverage and the small spot size (1–5 mm^2^). Industrial requirements for electronic and optoelectronic devices involve a thickness non-uniformity of less than 5% over 95% of the wafer surface, excluding the 5 mm edge [31]. To overcome this issue, advanced PLD systems employ target and substrate rotation, as well as a laser beam-synchronized droplet trap, preventing the macroparticles from arriving on the sample surface. This will ensure clean samples without PLD-specific droplets. Moreover, the laser beam movement on the target allows for the desired thickness to be achieved [31].

This study focuses on the fabrication of AZO thin films with high structural quality, good uniformity, and high optical transmittance, as well as low electrical resistivity, using a large-area PLD system. The main goal was to investigate the effect of deposition pressure on the physical properties of AZO films, together with a good uniformity of the deposition over a 4-inch area. Additionally, the results show that the specific requirements for TCO materials are fulfilled as the PLD-prepared AZO thin films exhibit transparency across the visible range, with a near-zero extinction coefficient (k), low electrical resistivity of 3.98 × 10^−4^ Ω cm, and a non-uniformity of less than 5% over 95% of the investigated area. Furthermore, these results closely align with the performance requirements for commercial TCOs and meet the industrial standards of large-area PLD technology.

## 2. Materials and Methods

The large-area AZO thin films were prepared using a PLD system capable of producing thin films up to 8 inches in size. AZO thin films were fabricated on 4-inch silicon wafers with an excimer laser (KrF-248 nm) and an Al-doped 2 wt.% ZnO (99.9%) target. Additionally, the PLD system features a droplet trap synchronized with the laser frequency, which helps prevent macroparticles from reaching the substrate, while enabling the production of uniform and low-roughness films. The schematic illustration of the PLD equipment, together with the description of its operation, is included in the study [32].

The study focuses on the impact of deposition pressure on the electrical and optical properties of AZO films. In this regard, the deposition was conducted in both high-vacuum and oxygen atmospheres at different deposition pressures. Furthermore, the deposition temperature was kept at 330 °C for all the prepared films, as this is considered optimal for these material [33,34]. An additional AZO sample was prepared at RT, but it was not considered within the article since it presented high porosity and very low charge mobility values. The experimental conditions are listed in Table 1.

Optical constants were determined using a Horiba Uvisel plus spectroellipsometer (France). Experimental measurements were performed at a light beam incidence angle of 70 degrees, over a spectral range of 0.6–6.5 eV (190–2100 nm) with a step of 0.02 eV. Electrical measurements were conducted with a cryogenic probe station controlled by a Lakeshore 8400 Series (USA), at room temperature and atmospheric pressure, using the four-probe method. Hall measurements were conducted to determine the carrier concentration, mobility, and resistivity of the samples in the Van der Pauw configuration.

## 3. Results

### 3.1. Morphological and Structural Characterization (SEM and XRD)

**Figure 1:**
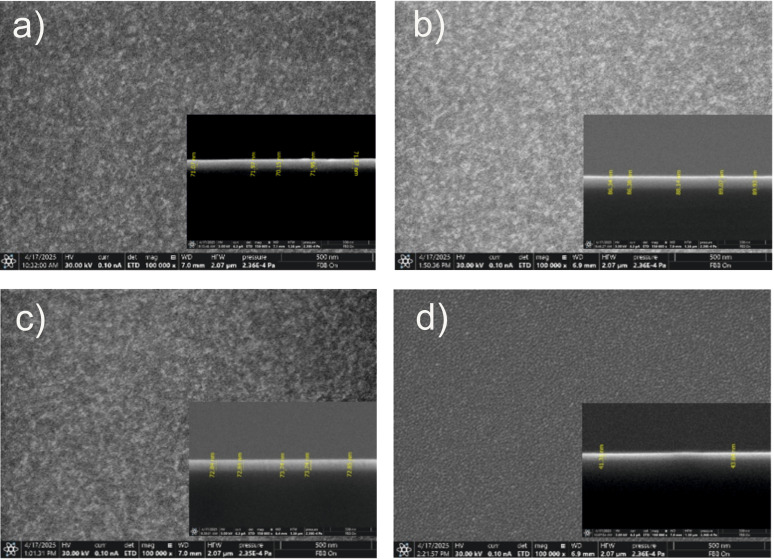
SEM images of AZO thin films prepared by PLD over a large area at different deposition pressures as follows: (**a**) under vacuum, (**b**) in 0.005 mbar O_2_, (**c**) in 0.01 mbar O_2_, and (**d**) in 0.1 mbar O_2_.

SEM images of the surface morphology of AZO thin films deposited by a PLD at different deposition pressures are illustrated in Figure 1. The top-view image confirms that AZO thin films were uniformly deposited, with a smooth surface free of droplets, cracks, or porosity [21]. The absence of droplets is primarily due to the “droplet trap”, which prevents the macroparticles from reaching the substrate surface. Additionally, the deposition pressure is considered the primary parameter affecting the surface of AZO films, since all the samples were deposited at the same substrate temperature, as shown in Table 1. Furthermore, it can be observed that the grain size increases as the deposition pressure increases. Moreover, the sample AZO4 prepared in 0.1 mbar O_2_ exhibits a different surface morphology, with more well-defined grains, compared to the samples prepared at low deposition pressures. The cross-sections reveal that the AZO thin films exhibit a columnar growth, with grains densely packed and without voids in the structure [35].

**Figure 2:**
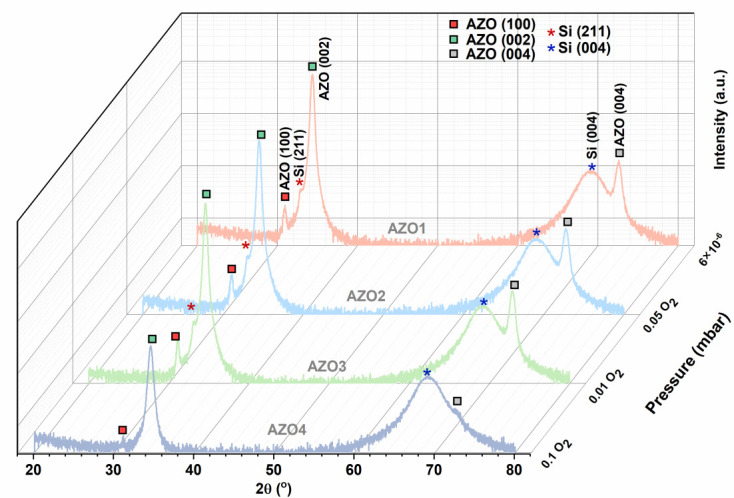
X-ray diffraction patterns of AZO thin films prepared by PLD over a 4-inch Si wafer.

Figure 2 shows the XRD pattern of AZO films prepared over a large-area Si wafer using PLD equipment. As can be observed, all AZO layers deposited at different pressures are polycrystalline, presenting Bragg peaks at 2θ = 31°, 34.4° and 72.6° corresponding to the (100), (002), and (004) reflections of the hexagonal ZnO structure. Additionally, all the AZO layers exhibit a strong (002) preferential orientation, with the c-axis perpendicular to the substrate surface and wurtzite hexagonal structure [36]. No diffraction peak of metallic Al is observed in AZO1, AZO2, AZO3, and AZO4 samples, suggesting the incorporation of Al ions within the ZnO lattice. Moreover, the crystallinity of AZO layers decreases as the deposition pressures increase. The AZO4 sample deposited in 0.1 mbar O2 exhibits the lowest intensity compared to the sample grown under vacuum conditions. Furthermore, the reduced crystallinity of the AZO4 sample is reflected in the electrical properties, resulting in a higher electrical resistivity [37]. This deterioration likely results from the background, which causes more scattering of the ablated species and reduces their kinetic energy [28].

### 3.2. Optical Characterization (SE)

**Figure 3:**
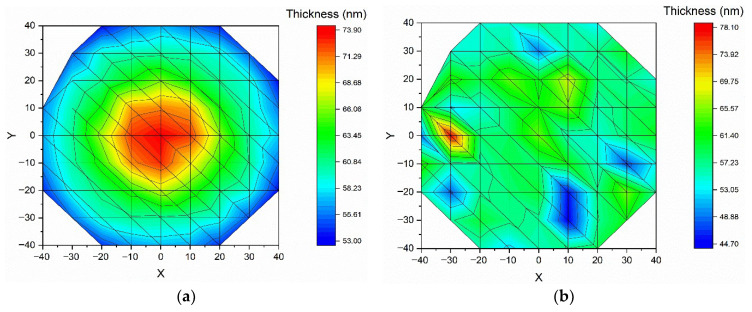

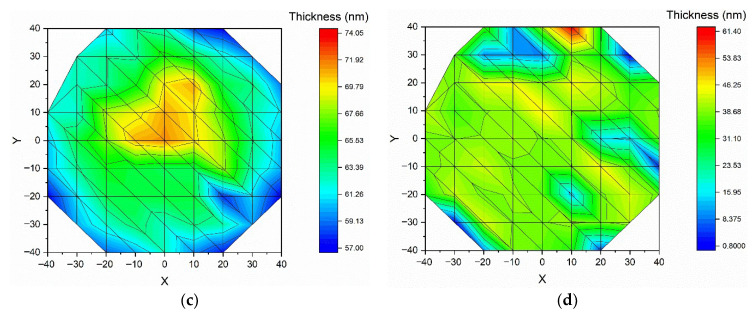
Thickness variation maps of AZO thin films prepared using PLD equipment on a 4-inch Si wafer at different deposition pressures: (**a**) under vacuum, (**b**) in 0.005 mbar O_2_, (**c**) in 0.01 mbar O_2_, and (**d**) in 0.1 mbar O_2_., measured with the SE technique in 68 points across the wafer.

The optical constants of AZO films, namely refractive index (*n)* and extinction coefficient (*k*), as well as their thickness (*t*), were determined using spectroscopic ellipsometry measurements. To assess this, an optical model was created to describe each layer of the samples, designed to represent the real AZO system. The model consists of a silicon substrate layer (0.65 mm), a native silicon oxide layer (3 nm), an AZO film layer of unknown thickness, and a top layer of unknown thickness representing the roughness of the films. For the roughness layer, the effective medium approximation (EMA) was applied, assuming a mixture of 50/50 volume fraction of voids and material. The parametrization used to evaluate the optical properties of the sample was based on the New Amorphous and Drude oscillators. Additionally, the Drude oscillator was introduced to describe the free-carrier contribution to the conductive behavior of the films. Further, the unknown parameters (*n*, *k*, *t*, and roughness) were determined by fitting the experimental data with the created model. Furthermore, the result of the fit was assessed using the Mean Squared Error (MSE), which represents the minimal difference between the model and the experimental data. Since the AZO samples were prepared on 4-inch Si wafers, ellipsometry measurements for thickness and uniformity evaluation were assessed at 68 points, starting from the center of the wafer and continuing to the edge. The thickness mapping across the wafer surface for all the AZO layers is shown in Figure 3.

According to the thickness maps presented in Figure 3, the AZO1 and AZO3 samples exhibit a higher thickness at the wafer center that slightly decreases toward the edge. In contrast, the AZO2 andAZO4, samples exhibit a more pronounced thickness variation at the wafer edges, while keeping a higher uniformity in the central region. Additionally, the standard deviation calculated for all the AZO samples, covering more than 96% of the surface, is displayed in Table 2.

**Figure 4:**
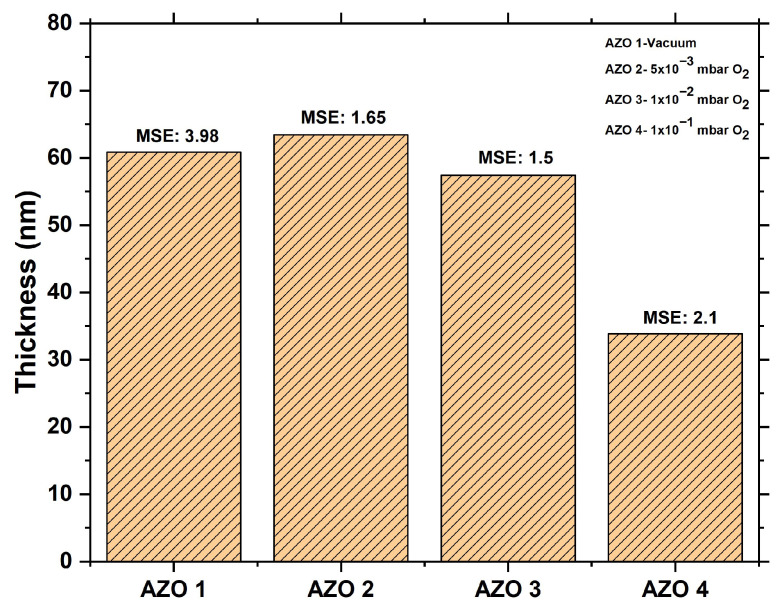
The average thickness calculated from the SE measurements across the 4-inch AZO wafer, along with the MSE for each sample.

As shown in Table 2, the σ of the AZO1 (HV), AZO2 (5 × 10^−3^ mbar O_2_), and AZO3 (1 × 10^−2^ mbar O_2_) samples prepared with the large-area PLD equipment is close to industry standards, which require a thickness variation of less than 5% from the center to the edge, excluding 5 mm from the wafer edge. The higher standard deviation value is observed for the AZO4 sample prepared at a deposition pressure of 0.1 mbar O_2_. This effect can be attributed to plasma confinement that occurs with increasing pressure, leading to a decrease in the thickness of the AZO4 film. Figure 4 shows the average thickness and MSE for each sample. All the MSE values were below 4, indicating that the chosen models are in good agreement with the physical system.

**Figure 5:**
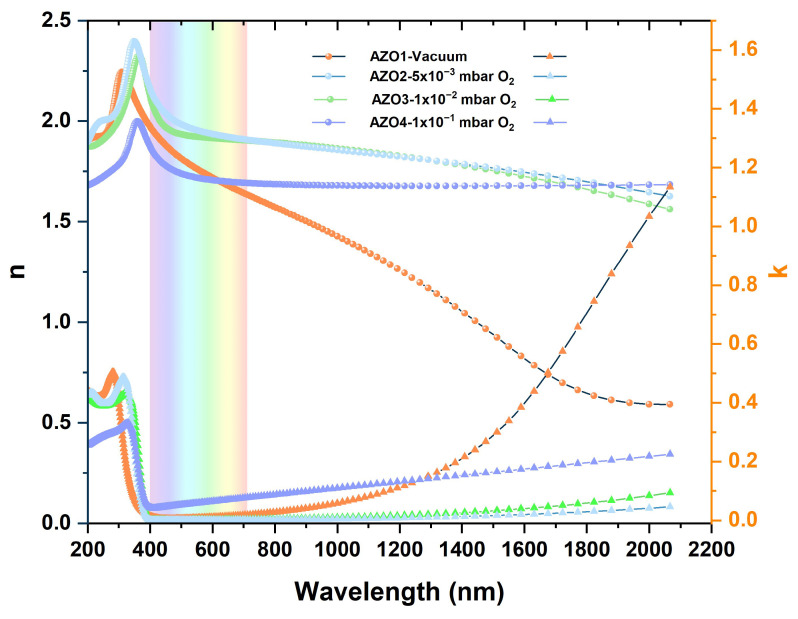
The dispersion of refractive index (n) and extinction coefficient (k) depending on wavelength for AZO films prepared by PLD and measured with SE at the center of the wafer.

Further, the optical constants are determined only for the central point of each sample and are displayed in Figure 5. In the UV region (200–320 nm), an abnormal dispersion is observed, where the refractive index (n) increases with wavelength, accompanied by a higher k value resulting from the strong absorption regime [38]. The fundamental absorption edge lies in the spectral range of 320–380 nm. In the visible range (400–800 nm), which corresponds to the weak absorption region, n exhibits normal dispersion, while k is close to zero. This behavior indicates that one of the essential requirements for TCO, transparency, is satisfied, making it suitable for optoelectronic applications. The refractive index given at 630 nm for each sample is as follows: 1.7—AZO1, 1.9—AZO2, 1.9—AZO3, 1.7—AZO4. These values are in concordance with those reported in the literature [39]. Additionally, the samples AZO2 and AZO3 present higher values of n compared with the other samples. The higher refractive index could suggest that these samples exhibit greater film density relative to the others [39]. However, the samples AZO1 exhibit a pronounced decrease in n with wavelength, accompanied by a strong increase in k. A similar behavior was observed in [18,40] for the AZO sample with 2% Al. This can be attributed to the incorporation of Al dopants in the ZnO lattice, which increases the free-carrier concentration and reduces the refractive index in concordance with the Drude model. However, native defects, such as oxygen vacancies or Zn interstitials, act as donor defects, thus increasing the number of free carriers in the sample. Furthermore, these defects may affect the structural properties of the sample, leading to a decrease in film density. At the same time, the increase in the free-carrier concentration will induce a decrease in the refractive index in the IR region, affecting the optical properties. Furthermore, the higher k observed in the IR region for AZO1 samples is in agreement with the assumption of increased carrier concentration, indicating a metallic behavior [40,41]. Additionally, our results are consistent with the study [42], where they found that the lowest resistivity is achieved for the sample that exhibits the highest extinction coefficient and lowest refractive index in the IR region. They attribute this behavior to the plasma resonance of free carriers.

**Figure 6:**
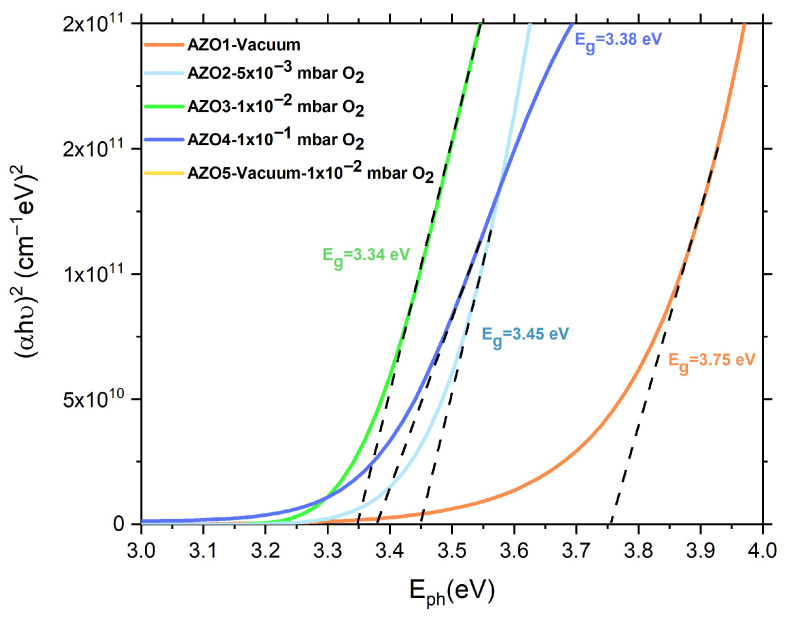
Variation in absorption coefficient (α) depending on wavelength, along with the insertion of the Tauc plot used for determining the bandgap energy (E_g_) for each sample.

Furthermore, the first step in determining the band gap (E_g_) energy was the calculation of the absorption coefficient (α) using the relation α = 4πk/λ, which links the absorption coefficient to the extinction coefficient determined through ellipsometry measurements (Figure 5). Next, the band gap energy was estimated using the Tauc method for direct transitions, based on the relation αhν = (hν − E_g_)^2^, where hμ represents the photon energy. Finally, E_g_ was extracted from the linear region of the (αhν)^2^ as a function of photon energy plot and can be seen in Figure 6. As we can observe in Figure 6, samples AZO3 and AZO4, prepared at deposition pressures of 0.01 mbar O_2_ and 0.1 mbar O_2_ exhibit band gap values close to the theoretical value of standard ZnO (E_g_ = 3.37 eV) [43], respectively, 3.34 eV for AZO3 and 3.38 eV for AZO4. In contrast, sample AZO2, prepared at a deposition pressure of 0.005 mbar O_2_, presents a slightly increased band gap of 3.45 eV. On the other hand, sample AZO1, deposited in HV, exhibited a strong increase in the band gap energy, specifically 3.75 eV. This behavior can be explained by the Burstein–Moss effect, which appears in degenerated semiconductors [11]. In degenerate AZO films, the Fermi level lies in the conduction band, and the optical transitions occur only between the valence band and the states in the conduction band that are above the Fermi level, leading to an apparent widening of the fundamental band gap. Further, this widening can be induced by an increase in free-carrier concentration, which is associated with defect formation, including oxygen vacancies (V_O_) and the substitution of Zn^2+^ ions with Al^3+^ ions, under the specific experimental conditions for the AZO1 sample.

### 3.3. Electrical Characterization

**Figure 7:**
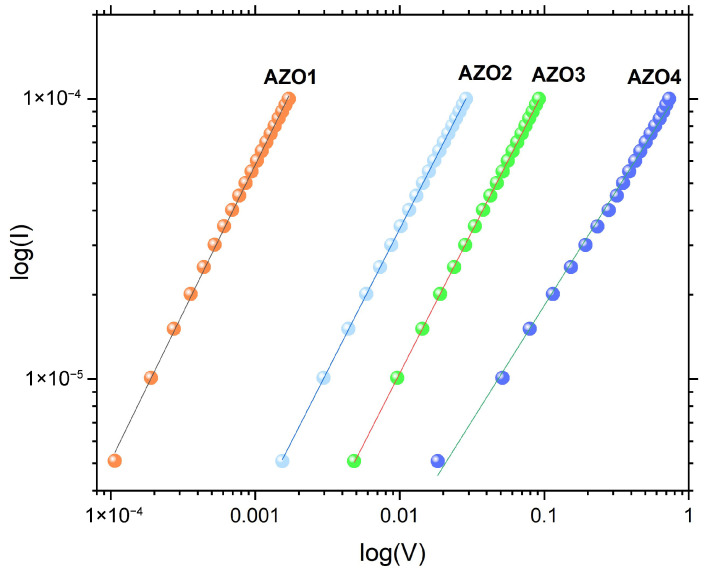
Log-log plot of I-V characteristics for AZO thin film deposition under different pressures, measured with the four-probe method.

The electrical properties of large-area AZO thin films deposited by PLD at different working pressures were assessed using the four-probe method. The I-V characteristic, measured at the center of 4-inch wafers, is presented in Figure 7. Since the electrical properties vary significantly with the deposition pressure, the I-V curves are displayed on a double-logarithmic scale. The slope of the I-V curves of all AZO layers was close to 1, indicating an ohmic behavior.

**Figure 8:**
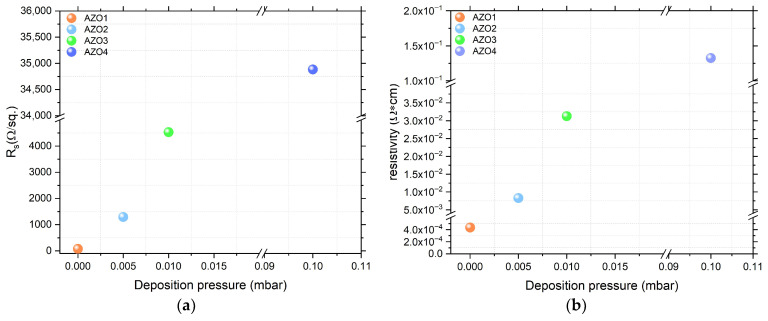
Variation in the Rs (**a**) and (**b**) resistivity as a function of deposition pressure for large-area PLD-grown AZO layers.

The variation in sheet resistance and resistivity assessed with the equation $Rs=\frac{\pi }{\ln\left(2\right)}\frac{U}{I}$ and *ρ* = $R_{s}$ t as a function of deposition pressure is shown in Figure 8a. In terms of sheet resistance, an available result is achieved for the sample AZO1 (72 Ω/sq.) prepared under HV conditions. In contrast, the sample AZO4 prepared in 0.1 mbar O_2_ exhibits an increase of three orders of magnitude in sheet resistance (34 kΩ/sq.) compared with the sample AZO1. Moreover, Rs increases with increasing deposition pressure, as can be observed in Figure 8a. Additionally, the electrical resistivity, displayed in Figure 8b, exhibits a similar behavior, with the lowest value of 4.33 × 10^−4^ Ω cm recorded for the sample AZO1 and reaching a maximum of 1.3 × 10^−1^ Ω cm for the sample AZO4. When discussing the electrical properties of AZO, it is important to specify that the conduction properties mainly arise from two principal mechanisms: (a) native defects, such as interstitial cations or anion vacancies, including interstitial zinc atoms (Zn_i_) and oxygen vacancies (V_o_), acting as donor-like defects and (b) doping by Al^3+^ ions, which donate one extra electron to the lattice, thereby improving the electrical conductivity [5]. According to this, the lower resistivity observed in samples prepared at low pressure may be attributed to oxygen deficiency. The absence of oxygen can induce oxygen vacancies (V_o_) or Zn_i_ interstitial atoms, which act as donor defects, significantly affecting the electrical properties of the AZO1 thin film. Moreover, the XRD analysis of the AZO1 sample indicates a slight shift in the 2θ diffraction angle associated with (002) reflection, which may result from the presence of such defects. Further, at high oxygen pressure, the resistivity trend increases. This behavior can be associated with the suppression of oxygen vacancies or Zn and Al interstitials, resulting in a decrease in free-carrier concentration [44]

**Figure 9:**
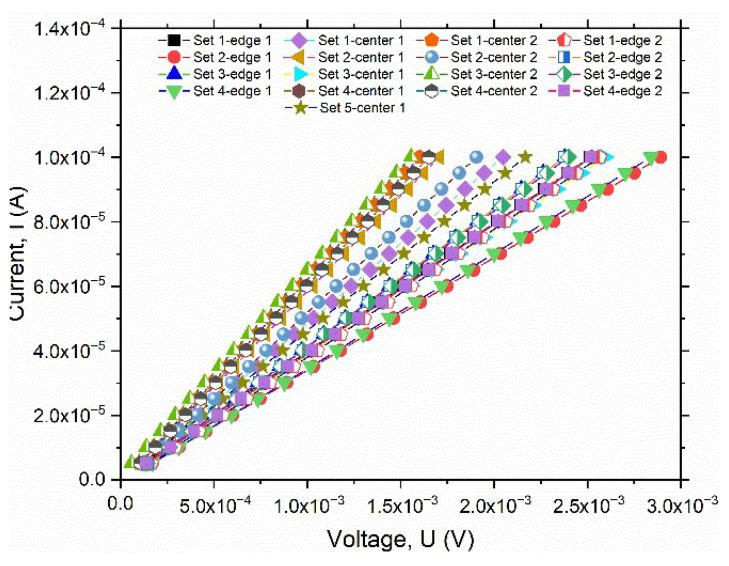
I-V characteristics measured across the AZO1 wafer.

Regarding the specific requirements for TCO, the optimal properties were achieved for the sample AZO1, prepared under vacuum conditions. This sample was further analyzed in detail. To evaluate the variation in electrical resistivity and sheet resistance across the wafer surface as a function of thickness, electrical measurements were performed at different points in the central region of the wafer. The I-V characteristics measured across the AZO1 wafer are shown in Figure 9.

**Figure 10:**
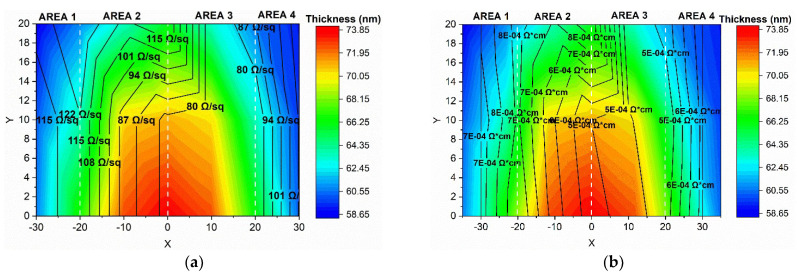
Contour plot of the variation in (a) sheet resistance Rs and (**b**) resistivity measured across the wafer, shown as a function of the corresponding thickness.

The contour plot in Figure 10 shows the variation in sheet resistance and resistivity with respect to the thickness of the measured area. As observed, the sample exhibits good uniformity in the measured quantities, with slightly increased values at the edge of the wafer. Considering the high requirements of scale-up TCO, the achievement of high uniformity in film thickness and electrical properties, combined with low resistivity, represents significant progress in the large-area deposition of AZO films, as well as an important step towards transferring PLD at an industrial scale.

The carrier concentration (*n*), Hall mobility (*µ*), and electrical resistivity (*ρ*) of the AZO sample prepared at different deposition pressures and measured at room temperature are listed in Table 3. Hall measurements performed in Van der Pauw configuration revealed that the sample AZO1 exhibits the lowest resistivity of 3.28 × 10^−4^ Ω cm. The resistivity increases with increasing deposition pressure, as observed in the four-probe measurements. Furthermore, the low electrical resistivity achieved for the AZO1 sample is attributed to a high carrier concentration of 1.05 × 10^21^ cm^−3^ and relatively high electrical mobility of 17.9 cm^2^/V s. Additionally, an increase in the deposition pressure leads to a reduction in both carrier concentration and mobility, as shown in Table 3. Moreover, the lowest carrier concentration of 7.7 × 10^18^ cm^−3^ is achieved for the sample AZO4 prepared at 0.1 mbar O_2_, which may result from either the suppression of native defects or the oxidation of Al atoms, leading to the formation of the Al_2_O_3_ phase. Nevertheless, the sample AZO1 exhibits an electrical mobility with an order of magnitude higher than that of the sample prepared at an oxygen pressure. Furthermore, the results obtained for the AZO sample prepared over a large area by PLD are comparable to those reported in the literature for ITO thin films.

**Figure 11:**
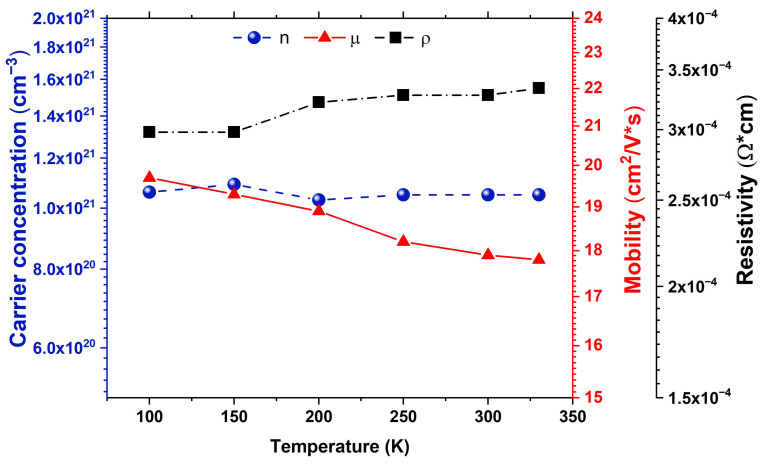
Hall measurements at the center of the AZO1 sample, performed in Van der Pauw configuration, as a function of temperature.

Figure 11 shows the Hall measurement of the carrier concentration (*n*), Hall mobility (*µ*) and resistivity (*ρ*) depending on temperature for the AZO1, measured at the center of the 4-inch wafer. Hall measurement revealed n-type conductivity over the entire temperature range. Moreover, the carrier concentration is independent of temperature, with a carrier concentration of ~1.05 × 10^21^ cm^−3^, indicating the degeneracy of the sample. Furthermore, the carrier concentration lies within the range of 10^21^ cm^−3^, representing the optimal concentration for such material in order to avoid unwanted optical absorption [48]. Hall mobility (*µ*) exhibits slight variation with the temperature, decreasing from 19.7 cm^2^/V s at 100 K to 17.9 cm^2^/V s at 300 K. Additionally, the measurement performed at 330 K revealed that the mobility (17.8 cm^2^/V s) does not change drastically with the temperature, which is an essential characteristic for the optimal performance of a TCO in the configuration of a device such as a photovoltaic cell or to be used for electromagnetic shielding applications (EMI). Additionally, the electrical resistivity exhibits a reduced value of 3.28 × 10^−4^ Ω cm, which is in agreement with the specific requirements for a TCO. Likewise, the electrical resistivity obtained from the four-probe measurement matches well with the one obtained from the Hall measurement in the Van der Pauw configuration. In addition, our results are consistent with those reported in the literature for AZO thin films prepared over a large area [4].

## 4. Conclusions

In this study, we investigated the effect of the deposition pressure on the physical properties of AZO thin films grown on a large area using an industrial PLD system. The deposited films exhibited high-quality morphology, characterized by the absence of droplets, cracks or porosity. Moreover, the optical characterization revealed an extinction coefficient (k) close to zero in the VIS region, confirming the transparency of the sample in the interest region of the electromagnetic spectrum. Additionally, the SE measurements confirm the thickness uniformity achieved on the 4-inch AZO thin films. Hall measurements validated the conductive behavior of the PLD-prepared samples, with the lowest resistivity and highest carrier concentration achieved for the sample prepared under HV conditions. Moreover, the large carrier mobility and the constant carrier concentration measured at 330 K confirm the stability of the sample at temperatures above RT. This result could suggest the possibility of integrating the AZO samples in devices that operate in harsh environments.

## Figures and Tables

**Table 1:** Experimental conditions for large-area PLD-grown AZO thin films on 4-inch Si substrate.

Sample	T (°)	P_dep_ (mbar)	Fluence (J/cm^2^)	Repletion Rate (Hz)	T-S Distance (cm)	Nr Laser Pulses
AZO1	330	Vacuum (6 × 10^−6^)	2	10	6	10.000
AZO2		5 × 10^−3^				
AZO3		1 × 10^−2^				
AZO4		1 × 10^−1^				

**Table 2:** The standard deviation (σ) calculated in 1σ for all the AZO layers.

σ (standard deviation)	**AZO1**	**AZO2**	**AZO3**	**AZO4**
	5.5	4.6	4	12.8

**Table 3:** Carrier concentration (*n*), Hall mobility (*µ*), and electrical resistivity (*ρ*) of the AZO sample prepared at different deposition pressures and measured at room temperature, compared with the literature results reported on ITO thin films prepared with PLD.

**Sample**	**P_dep_** **(mbar)**	***n*** **(cm^−3^)**	***µ*** **(cm^2^/V s)**	***ρ*** **(Ω cm)**	vs. ITO	***ρ*** **(Ω cm)**	***n*** **(cm^−3^)**	***µ*** **(cm^2^/V s)**	**Ref.**
AZO1	HV	1.05 × 10^21^	17.9	3.28 × 10^−4^		4 × 10^−4^	-	-	[45]
AZO2	0.005 O_2_	2.72 × 10^20^	4	5.57 × 10^−3^		-	4 × 10^20^	-	[46]
AZO3	0.01 O_2_	1.01 × 10^20^	2.47	2.49 × 10^−2^		2 × 10^−4^	1.4 × 10^21^	27	[47]
AZO4	0.1 O_2_	7.7 × 10^18^	7	1.1 × 10^−1^		-	-	-	-

## Data Availability

The original contributions presented in this study are included in the article. Further inquiries can be directed to the corresponding author.
